# Factors Associated with Severity of Post-Intubation Cicatricial Laryngeal Stenosis in Children: A Retrospective Study

**DOI:** 10.3390/jcm15093342

**Published:** 2026-04-27

**Authors:** Nazym Sagandykova, Madina Baurzhan, Aigerim Mashekova, Yerkin Abdildin, Makhabat Baimurzayeva, Olzhas Mukhmetov, Eddie Yin Kwee Ng, Sayagul Kairgeldina

**Affiliations:** 1 Corporate Fund “University Medical Center”, Astana 010000, Kazakhstan; doctor.ent.alm@gmail.com (N.S.); mbaymurzaa@gmail.com (M.B.); 2 Scientific Research Institute of Balneology and Medical Rehabilitation, Astana 010000, Kazakhstan; madina_baurzhan@mail.ru (M.B.); sanborovoe@mail.ru (S.K.); 3 Department of Mechanical and Aerospace Engineering, School of Engineering and Digital Sciences, Nazarbayev University, Astana 010000, Kazakhstan; yerkin.abdildin@nu.edu.kz (Y.A.); olzhas.mukhmetov@nu.edu.kz (O.M.); 4 School of Mechanical and Aerospace Engineering, Nanyang Technological University, Singapore 639798, Singapore

**Keywords:** laryngeal stenosis, intubation, stenosis severity, risk factors, pediatric airways

## Abstract

**Background.** Post-intubation cicatricial laryngeal stenosis (PICLS) represents one of the most severe long-term complications of pediatric airway management. By systematically analyzing clinical and procedural variables across different grades of PICLS, this study addresses a critical gap in pediatric airway research and provides clinically relevant descriptive data on stenosis severity. **Materials and methods.** A retrospective single-center case-series study was conducted and included pediatric patients (0–18 years) treated for PICLS at a tertiary referral pediatric otolaryngology center between 2016 and 2024. Spearman correlation and multiple regression analyses were used to evaluate possible associations between clinical factors and stenosis grade. **Results.** Among 172 children with PICLS, severe forms of stenosis (Grades 3–4) were observed in 37.2%, with predominant subglottic localization (85.3%). Age at primary intubation (*p* = 0.02) and the type of intubation (emergency/elective; *p* = 0.04) were the only variables significantly associated with stenosis severity in this cohort, whereas sex, reintubation, comorbidities, and delivery-related factors showed no significant associations. Mild stenosis (Grades 1–2) more frequently followed intubation for elective surgery and infections, whereas severe stenosis was more commonly associated with intubation due to central nervous system pathology and infections. **Conclusions.** Age at primary intubation and the type of intubation (emergency/elective) were associated with stenosis severity in this cohort. These findings should be interpreted in light of the retrospective case-series design and the absence of a control group, but they may contribute to improved clinical characterization of PICLS severity in children.

## 1. Introduction

Laryngeal cicatricial stenosis in children is a serious condition that significantly reduces their quality of life and sometimes leads to disability. Life-saving intubation can lead to post-intubation cicatricial laryngeal stenosis [1,2,3]. According to data from various clinics [4,5], approximately 3% of children who have undergone intubation develop airway stenosis.

Cicatricial laryngeal stenosis is caused by damage of the mucosal layer during tube insertion, as well as contact with the inflated cuff [6,7]. This leads to the development of mucosal inflammation and fibrotic changes in the upper respiratory tract [8,9].

Despite modern anesthetic standards, there is a risk of developing laryngeal stenosis after intubation. This problem is relevant in both countries with developed healthcare systems [10] and in those with limited access to medical resources [11,12].

Among the known risk factors for the development of post-intubation laryngeal stenosis (PICLS) are the child’s age at the time of primary laryngeal intubation [13,14,15,16], especially infancy [5]; gestational age [17,18]; the duration of intubation [5]; the number of intubations [19]; the diameter of the endotracheal tube [4,19,20]; and the tube length [21]. Some sources also indicate a link between intubation and the development of stenosis in patients with central nervous system (CNS) pathology and congenital heart disease [22,23].

Veder et al. (2024) [24] indicated that only sedation level and gastroesophageal reflux disease (GERD) are considered risk factors, while other proposed factors had low evidence. The literature has primarily examined new-onset stridor after intubation, which is not the only clinical manifestation of laryngeal stenosis. However, tissue changes in the form of fibrous laryngeal stenosis in children have not been considered separately. Accordingly, little attention has been paid to specific risk factors for cicatricial stenosis of the larynx in children. Only limited data on risk factors according to the degree of laryngeal stenosis are available [25].

The aim of this retrospective case series was to provide a clinical and demographic description of children with PICLS and to examine severity-associated clinical patterns within this cohort.

## 2. Materials and Methods

### 2.1. Study Design and Settings

This study is a retrospective case-series analysis conducted at the Department of Pediatric Surgery (Head-Neck Program) CF “University Medical Center”, a tertiary referral pediatric otolaryngology department in Astana, Kazakhstan. The study included children hospitalized between January 2016 and December 2024 due to PICLS diagnosis and/or those present for surgical treatment, including endoscopic evaluation of the larynx, which was performed in young children under general anesthesia. Patients with acquired cicatricial stenosis of the larynx were carefully selected, excluding other types of stenosis that can also occur after intubation (formations, vocal fold paresis, etc.).

Inclusion criteria: age between 0 and 18 years; a history of endotracheal intubation for any reason (e.g., surgery, resuscitation, infectious diseases, trauma, etc.); and endoscopic confirmation of fibrotic changes in the larynx with stenosis. Written informed consent for participation and data processing was obtained from patients or their parents/legal guardians (for minors).

Exclusion criteria: missing data on the degree of stenosis; primary congenital laryngeal stenosis or other congenital malformations of the larynx; and laryngeal stenosis caused by factors unrelated to intubation, such as autoimmune or systemic diseases (e.g., Wegener’s granulomatosis, sarcoidosis), laryngeal or tracheal malignancies, or post-radiation fibrosis, trauma, chemical burns, and tracheal stenosis.

In addition, we excluded patients without verifiable documentation of intubation (e.g., cases where intubation is not recorded in their medical history despite subsequent development of symptoms).

Patients who previously underwent reconstructive surgery on the larynx were also excluded; acute upper respiratory tract infections and other conditions that may temporarily obscure the severity of stenosis; refusal to participate in the study by the parents or legal guardians; or absence of written informed consent in the medical records were also factors for patient exclusion.

### 2.2. Data Collection

Data was manually extracted from hospital medical records into Excel spreadsheets.

The following variables were recorded: age; sex; stenosis severity grade according to the Myer–Cotton classification (Grades 1–4); localization of stenosis (supraglottic, glottic, subglottic, or combined); whether intubation was emergency or planned; presence of comorbidities (respiratory; endocrinological; infection (urinary, neurological, respiratory/congenital, cardiovascular/congenital, gastro/congenital, excretory/congenital, neurological/congenital, or combined/congenital); a history of reintubation (yes or no); presence of a T-tube or tracheostomy (yes or no); the type of delivery (vaginal or cesarean section); gestational age at birth (preterm or full-term); the type of residence (urban or rural); and the level of the healthcare facility where intubation was performed (primary, secondary, or tertiary).

The reasons for Intubation, such as planned surgery and neonatal distress syndrome, were also analyzed. Some variables had missing data; analyses were performed using the available cases.

Data were collected and stratified by three ENT doctors and double-checked by a senior doctor and a data analyst. All patients’ names were numbered in the order of their arrival to the department (e.g., 2016-15, 2016-16, etc.).

### 2.3. Methods of Data Processing

Multiple regression and Spearman correlation analyses were performed. The Data Analysis ToolPak in Microsoft Excel was used for statistical analysis.

## 3. Results

**Figure 1:**
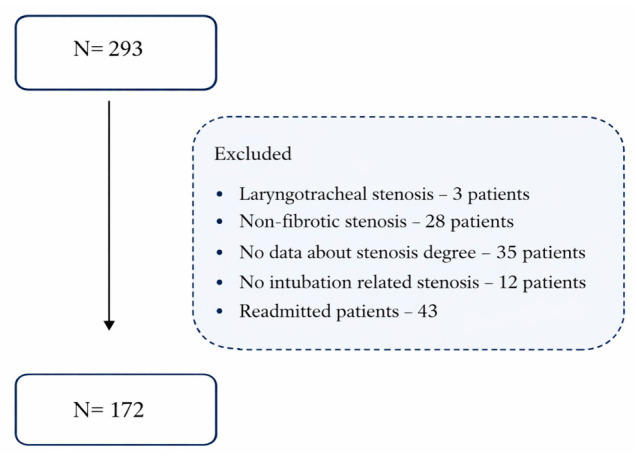
Enrollment of patients with PICLS.

In total, 293 children with laryngeal stenosis were hospitalized in the Head and Neck Department during the 2016–2024 period. Only 172 met the selection criteria, namely those with PICLS. A total of 121 patients were excluded for various reasons: other causes of upper airway narrowing (laryngeal papillomatosis or laryngeal hemangioma); stenosis due to factors other than intubation (burn or laryngeal trauma); repeated hospitalizations of patients (some patients were reported several times); and a combination with tracheal stenosis. For some patients, the PICLS grade was not specified in their medical records; therefore, they were also excluded from the study (Figure 1). Furthermore, missing data was found in some categories, so the number of patients varied from 170 to 172 across variables.

### 3.1. Common Characteristics for Children with PICLS

Table 1 summarizes the baseline clinical and demographic characteristics of the pediatric PICLS cohort. The study population showed a slight male predominance, and most children were intubated during infancy, with 70.8% undergoing primary intubation within the first year of life, while the median age at the time of initial intubation was 6.2 months. Overall, the study cohort was characterized by a predominance of milder degrees of stenosis, subglottic lesions, and emergency intubation. A significant proportion of children had comorbidities and a history of tracheostomy. The majority of patients were also born at full term via vaginal delivery and resided in urban areas.

### 3.2. Stenosis Severity by Clinical Factors

**Figure 2:**
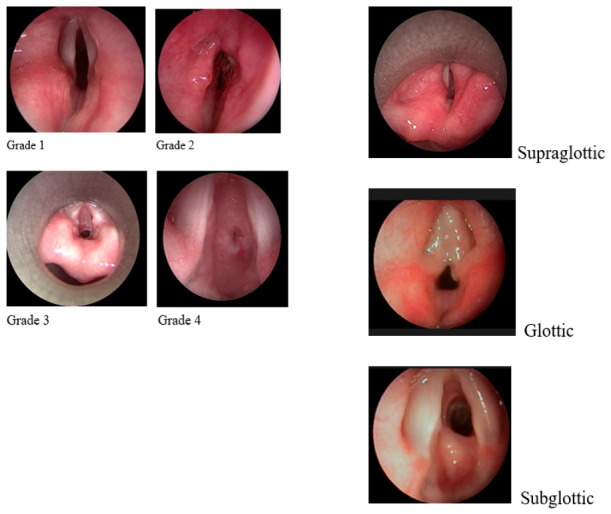
Endoscopic picture of the PICLS children’s larynx.

Patients were classified into four grades of laryngeal stenosis according to the Myer–Cotton classification (Figure 2). Combined types of stenosis were classified, such as supraglottic–glottic, glottic–subglottic, etc. Tracheal stenosis was not included, as most of the cases were associated with the installation of a tracheostomy tube.

Among the identified indications for intubation, elective surgery and infection were more commonly observed in children with milder stenosis (Grades 1–2), whereas CNS pathology accounted for a larger share of severe stenosis, particularly Grade 4. Infection-related intubation remained frequent across all stenosis grades. Subglottic localization predominated in all severity groups, while combined forms were less common. In addition, children without comorbidities constituted a larger proportion of Grade 4 stenosis, whereas children with comorbidities were more frequently represented in milder grades (Table 2).

### 3.3. Clinical Differences Between PICLS Patients with and Without Comorbidities

**Figure 3:**
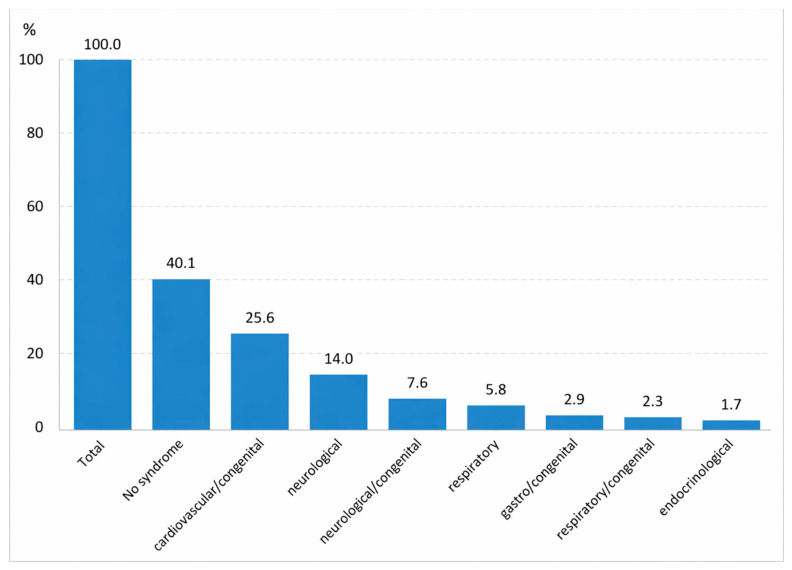
Overall comorbidity shares (%) in the pediatric PICLS population (n = 172).

A comparison of children with and without comorbidities revealed several descriptive differences (Table 3). In both groups, the majority of patients were intubated within the first year of life, and subglottic stenosis was the predominant localization. Children with comorbidities more frequently underwent intubation for elective surgery, whereas children without comorbidities more often underwent emergency intubation (Figure 3). Neonatal causes and airway obstruction were relatively more frequent in the non-comorbidity group, while CNS pathology and infection were common in both groups. Sex distribution, reintubation history, and prior tracheostomy were broadly similar between the two cohorts.

### 3.4. Correlation and Regression Analysis of Stenosis Grade

According to multiple regression analysis (Table 4), statistically significant associations with the degree of stenosis were found for age at primary intubation (*p* = 0.02) and the type of intubation (emergency/elective; *p* = 0.04). The remaining variables did not show a statistically significant association with the degree of stenosis. However, the overall model did not achieve statistical significance (*p* = 0.07) and was characterized by limited explanatory power (R^2^ = 0.118; adjusted R^2^ = 0.051); therefore, the obtained results should be interpreted with caution, as it is exploratory.

According to the correlation analysis (Table 5), the degree of stenosis demonstrated only weak associations with age at initial intubation, the type of intubation, and reintubation, while no significant correlations were found for the other clinical and demographic variables studied. Overall, the relationships between the degree of stenosis and the variables included in the analysis were weak.

Key Findings

1. Children under one year old have often been intubated (70.8%);
2. Grade 1 PICLS (39.5%) and subglottic localization prevailed (85.3%);
3. In 82.6% of cases, intubation was performed on an emergency basis, and only 31.6% of patients had a history reintubation;
4. Mild forms of stenosis (Grades 1 and 2) were more frequently observed after intubations for planned surgeries and infections, whereas severe forms (Grades 3 and 4) were more commonly observed after intubations for CNS pathologies and infections;
5. Thirteen (65%) patients with Grade 4 stenosis had no comorbid diseases.

## 4. Discussion

PICLS in children remains one of the most severe and clinically significant complications of intubation, often leading to persistent respiratory distress and disability. Although PICLS develops in a relatively small proportion of intubated patients [4], iatrogenic stenosis is characterized by a more aggressive course and worse outcomes compared to other etiologies [26].

This study is the largest single-center study of pediatric PICLS to date (n = 172) and provides a stratified analysis of the clinical and demographic characteristics of children with mild (Grades 1–2) and severe (Grades 3–4) PICLS, as well as possible associations with stenosis severity. Unlike existing publications, which primarily focus on adult patients [27], specific etiologies of stenosis in children [28], or small sample sizes [29], the current study encompasses wide clinical and demographic variability and allows for comparison of clinical features between severity groups.

The present study includes data from 2016 to 2024, in which 62.8% of patients had mild stenosis (Grade 1 and 2), while severe stenosis (Grade 3 and 4) was detected in 37.2% of children. This distribution of the ratio of mild to severe stenosis is generally comparable to the data of Redondo-Sedano et al. (2019) [25], who reported 61.5% mild and 38.4% severe stenosis. However, in their study, only 33% of patients had stenosis associated with previous intubation, whereas in the current cohort, all cases occurred post-intubation, highlighting the specificity and more homogeneous etiology of the study group.

In the present study, the collected demographic data were generally consistent with the results of other studies. The cohort showed a slight predominance of male patients (59.3%), which is comparable to the data of Shinn et al. (2019) [30]. Furthermore, 85.3% of cases of stenosis were located in the subglottic space, which is also reflected in studies describing the predominant development of subglottic stenosis after intubation and tracheostomy [5,31,32]. The predominance of subglottic localization of stenosis may be associated with the anatomical and functional features of this zone, as well as with the mechanical impact of the endotracheal tube and its cuff on the mucous membrane of the subglottic region, which potentially contributes to the development of inflammation with subsequent stenosis [33,34].

At the same time, our sample also revealed several differences from published data. Most international studies describe stenosis in children primarily as a complication of planned intubation [5,18], whereas in our cohort, 82.6% of patients underwent emergency intubation. This likely reflects differences in healthcare systems and neonatal distress syndrome management practices, as well as resuscitation protocols.

According to several studies, repeated intubation may increase the risk of laryngeal injury [35,36]. However, in our cohort, the majority of children (68.4%) only underwent one intubation event. This suggests that clinically significant cicatricial laryngeal stenosis may occur even after a single intervention. However, this finding should be viewed with caution, as several important procedural characteristics were not available in the present study, including intubation duration, tube size, and cuff parameters, which could potentially have a significant impact on the severity of airway injury.

In the present study, stenosis was predominantly detected in infants (70.8%), and the median age at initial intubation was 6.2 months. This is considerably lower than the 23 months reported by Percul et al. [37]. This difference may reflect both the structural features of our cohort and the increased vulnerability of the airway in infants. In early childhood, the subglottic region of the larynx is anatomically narrower and more sensitive to mechanical stress, so even relatively limited intubation trauma can lead to scarring. This may partially explain why infants constituted the majority of the study cohort.

The collected data suggest that a younger age at primary intubation was associated with age at primary intubation (*p* = 0.02) and the type of intubation (emergency/elective; *p* = 0.04), while the role of traditional clinical factors remains less clear and requires further investigation in prospective studies. These results should be interpreted with caution given the descriptive nature of the study. The results of other studies are inconclusive. Several studies have found no effect of age or other potential risk factors—such as infection, tube size, intubation duration, or other procedural characteristics—on the likelihood of developing stenosis [30,38,39]. Exceptions included the level of sedation during intubation [40] and the presence of gastroesophageal reflux disease [41], which were considered possible predisposing factors [24].

The clinical and etiological profile was heterogeneous: mild forms of stenosis (Grades 1 and 2) occurred more often after intubation for elective surgeries and infections, while severe forms (Grades 3 and 4) predominated after intubation for CNS pathology as well as for infections. In the literature, causes of intubation are mainly described in a general context: for example, with neonatal distress syndrome, stenosis developed in more than 80% of cases [42], and mechanical ventilation for >21 days was associated with subglottic stenosis in 54.17% of children, of which complex forms occurred in only 3% [27]. The list of indications for intubation (elective surgeries, neonatal disorders, trauma, neurological causes, etc.) was systematized by Veder et al. (2020) [28]. Separately, it has been shown that the incidence of severe post-intubation laryngeal lesions in COVID patients reached 45% [43]. Unlike most studies, which focus on a single cause of intervention, the present analysis compares different etiologies and demonstrates their association with stenosis severity.

Despite a significant body of work on perioperative airway injury prevention [44], the pathogenesis of cicatricial remodeling of the larynx in some patients remains poorly understood [45]. An additional methodological limitation of the literature is that cicatricial fibrous stenosis is often analyzed in conjunction with other causes of stenosis (tumors, granulation tissue, and vocal fold dysfunction), complicating the interpretation of risk factors.

Against this background, the cohort demonstrates a different comorbidity distribution: 40.1% of children had no comorbidities, whereas in some series, the comorbidity rate in patients with PICLS was as high as 88% [29]. This discrepancy highlights the heterogeneity of the populations and the possible influence of organizational factors within the healthcare system.

In the present study, clinical and demographic characteristics, as well as individual parameters related to intubation (age at primary intubation, indications for intubation, frequency of intubation, etc.), were analyzed in children with verified PICLS while taking into account the degree of stenosis, an aspect that is relatively rarely represented in pediatric studies. According to multiple regression analysis, a statistically significant association with the degree of stenosis was found only for age at primary intubation (*p* = 0.02) and the type of intubation (emergency/elective; *p* = 0.04), while no consistent associations were obtained for the other variables studied. In a similar study, Cakir et al. (2020) [42] did not find an association between the duration of intubation and the severity of stenosis. This may likely be due to the absence of some important procedural characteristics (intubation duration, tube diameter and length, and cuff pressure), as well as the limited ability of the available clinical parameters to fully reflect the severity of subsequent cicatricial remodeling.

## 5. Limitations and Future Directions

This study has several limitations. First, its retrospective, single-center design limits the completeness and standardization of the baseline data. Second, selection bias is possible, as this study was conducted at a specialized center where children with clinical manifestations of laryngeal stenosis are hospitalized for both diagnostic and therapeutic purposes. Third, this study did not include a control group of intubated children without stenosis, which precludes a strict assessment of risk factors for the development of PICLS. Rather, it only allows for a description of the clinical and demographic characteristics of the cohort and possible associations with the degree of stenosis. An additional limitation is the absence or incompleteness of several important procedural data in the medical records, including intubation duration, tube size, cuff pressure, and other parameters that could potentially influence the severity of airway damage. Furthermore, the results of the multiple regression model should be interpreted with caution given the exploratory nature of the analysis and the limited set of available variables. At the same time, this study is valuable as one of the largest single-center descriptive studies of children with PICLS and, to our knowledge, one of the few that has included an analysis that takes into account the degree of stenosis. Future multicenter prospective studies would be advisable, including a more comprehensive set of procedural intubation characteristics and, if possible, a control group, to more accurately assess the factors associated with the severity and development of PICLS.

## 6. Conclusions

This study characterized the clinical and demographic profile of children with PICLS. Grade 1 stenosis and subglottic localization were the most common findings in the cohort. Milder forms of stenosis were more frequently observed after intubation for elective surgery and infection, whereas more severe forms were more common among children intubated due to central nervous system pathology and infection. Age at primary intubation and the type of intubation (emergency/elective) were the only variables significantly associated with stenosis grade; however, these findings should be interpreted cautiously in light of the descriptive and exploratory nature of this study. Overall, these results provide clinically relevant descriptive data and may serve as a basis for further studies of factors associated with PICLS severity in children.

## Figures and Tables

**Table 1:** Baseline clinical and demographic characteristics of pediatric patients with PICLS.

Characteristics	Overall, *n* (%)
Sex:	172
Male	102 (59.3%)
Female	70 (40.7%)
Age at primary intubation:	171
Infants (≤12 months)	121 (70.8%)
children (1–12 years)	46 (26.9%)
adolescent (13–18 years)	4 (2.3%)
Grade of stenosis:	172
1	68 (39.5%)
2	40 (23.3%)
3	45 (26.2%)
4	19 (11%)
Localization:	171
Supraglottic	1 (0.7%)
Glottic	5 (2.9%)
Subglottic	146 (85.3%)
Combined	19 (11.1%)
Comorbidities:	172
yes	103 (59.9%)
no	69 (40.1%)
Intubation emergency:	172
emergency	142 (82.6%)
planned	30 (17.4%)
Reintubation:	171
yes	54 (31.6%)
no	117 (68.4%)
T-tube:	172
yes	2 (1.2%)
no	170 (98.8%)
Tracheostomy:	172
yes	108 (62.8%)
no	64 (37.2%)
Gestational age at delivery:	170
Preterm delivery	30 (17.6%)
Term delivery	140 (82.4%)
Delivery type:	170
vaginal	135 (79.4%)
cesarean section	35 (20.6%)
Residence:	171
urban	109 (63.7%)
rural	62 (36.3%)

**Table 2:** Patient characteristics stratified by laryngeal stenosis grade in children.

-	Grade 1 (*n* = 68; 39.5%)	Grade 2 (*n* = 40; 23.3%)	Grade 3 (*n* = 40; 26.2%)	Grade 4 (*n* = 24; 11%)
Sex (*n* = 172)				
Male (*n* = 102)	43 (63.2%)	22 (55%)	25 (55.5%)	12 (63.2%)
Female (*n* = 70)	25 (36.8%)	18 (45%)	20 (44.5%)	7 (36.8%)
%	100%	100%	100%	100%
Age at primary intubation (*n* = 171)				
Infants (0 to ≤ 12 month(s)	54 (79.4%)	25 (62.5%)	32 (72.7%)	10 (52.6%)
Children (1–12 years)	14 (20.6%)	15 (37.5%)	11 (25.0%)	6 (31.6%)
Adolescents (13–18 years)	0	0	1 (2.3%)	3 (15.8%)
%	100%	100%	100%	100%
Reintubation (*n* = 171)				
Yes	19 (27.9%)	11 (27.5%)	18 (41%)	6 (31.5%)
No	49 (72.1%)	29 (72.5%)	26 (59%)	13 (68.5%)
%	100%	100%	100%	100%
Delivery time (*n* = 170)				
Preterm delivery	9 (13.4%)	11 (27.5%)	6 (13.6%)	4 (21.05%)
Term delivery	58 (86.6%)	29 (72.5%)	38 (86.4%)	15 (78.95%)
%	100%	100%	100%	100%
Delivery type (*n* = 170)				
Vaginal delivery	54 (79.4%)	30 (76.9%)	37 (84.1%)	14 (73.7%)
Cesarean section	14 (20.6%)	9 (23.1%)	7 (15.9%)	5 (26.3%)
%	100%	100%	100%	100%
Residence (*n* = 171)				
Urban residence	45 (67.2%)	27 (67.5%)	27 (60%)	10 (52.6%)
Rural residence	22 (32.8%)	13 (32.5%)	18 (40%)	9 (47.4%)
%	100%	100%	100%	100%
Indications for intubation (*n* = 171)				
Elective surgery	26 (38.2%)	6 (15.0%)	9 (20.0%)	1 (5.3%)
Infection	18 (26.5%)	23 (57.5%)	16 (35.6%)	4 (21.1%)
CNS pathology	11 (16.2%)	5 (12.5%)	11 (24.4%)	11 (57.9%)
Mechanical obstruction	6 (8.8%)	3 (7.5%)	7 (15.6%)	2 (10.5%)
Neonatal respiratory pathologies	6 (8.8%)	3 (7.5%)	2 (4.4%)	1 (5.3%)
No data	1 (1.5%)			
	100%	100%	100%	100%
Localization (*n* = 171)				
Supraglottic, (*n* = 1)	1 (1.5%)	0	0	0
Glottic, (*n* = 5)	2 (2.9%)	2 (5%)	1 (2.2%)	0
Subglottic (*n* = 146)	60 (88.2%)	33 (82.5%)	36 (80.0%)	18 (90%)
Combined (*n* = 19)	5 (7.4%)	5 (12.5%)	8 (17.8%)	2 (10%)
%	100%	100%	100%	100%
History of tracheostomy (*n* = 171)				
Yes	42 (61.8%)	19 (47.5%)	31 (68.9%)	17 (85%)
No	26 (38.2%)	21 (52.5%)	14 (31.1%)	3 (15%)
%	100%	100%	100%	100%
Intubation clinic’s level (*n* = 171)				
Primary	23 (33.8%)	13 (32.5%)	17 (37.8%)	4 (20%)
Secondary	26 (38.2%)	21 (52.5%)	25 (55.6%)	14 (70%)
Tertiary	19 (28%)	5 (12.5%)	3 (6.6%)	2 (10%)
No data	0	1 (2.5%)	0	0
Comorbidities (*n* = 172)				
Patients with comorbid.	47 (69.1%)	23 (57.5%)	26 (57.8%)	7 (35%)
Patients without comorbid.	21 (30.9%)	17 (42.5%)	19 (42.2%)	13 (65%)
%	100%	100%	100%	100%

**Table 3:** Comparison characteristics between cohorts of patients with and without comorbidities.

		Patients with Comorbid. (n = 103)	Patients Without Comorbid. (n = 69)
Sex	Male	61 (59.2%)	41 (59.4%)
	Female	42 (40.8%)	28 (40.6%)
		*100%*	*100%*
Age at primary int.	infants (≤12 months)	77 (74.7%)	44 (63.7%)
	children (1–12 years)	26 (25.3%)	20 (28.9%)
	adolescent (13–18 years	0	4 (5.7%)
No data		0	1 (1.7%)
		100%	100%
Localization	Supraglottic	1 (1.0%)	0 (0.00%)
	Glottic	2 (1.9%)	3 (4.3%)
	Subglottic	88 (85.4%)	58 (84.1%)
	Combined	12 (11.7%)	8 (11.6%)
		100%	100%
Reason of intubation	CNS pathology	22 (21.4%)	16 (23.2%)
	Elective surgery	34 (33.0%)	9 (13.0%)
	Infection	36 (35.0%)	25 (36.2%)
	Neonatal causes	4 (3.8%)	7 (10.1%)
	Obstruction	7 (6.8%)	11 (16.0%)
	No data	0 (0.0%)	1 (1.5%)
		100%	100%
Reintubation	Yes	33 (32.1%)	21 (30.4%)
	No	70 (67.9%)	47 (68.1%)
	No data	0 (0.00%)	1 (1.5%)
		100%	100%
Emer/planned int	Emergency	79 (76.7%)	63 (91.3%)
	Planned	24 (23.3%)	6 (8.7%)
		100%	100%
History of tracheostomy	Yes	67 (65.0%)	41 (59.4%)
	No	36 (35.0%)	28 (40.6%)
		100%	100%

**Table 4:** Multiple regression analysis.

*Regression Statistics*								
Multiple R	0.343010425							
R Square	0.117656151							
Adjusted R Square	0.050719721							
Standard Error	1.04056021							
Observations	157							
ANOVA								
	df	SS	MS	F	Significance F			
Regression	11	20.94	1.90	1.76	0.07			
Residual	145	157.00	1.08					
Total	156	177.94						
	Coefficients	Standard Error	t Stat	*p*-value	Lower 95%	Upper 95%	Lower 95.0%	Upper 95.0%
Intercept	1.22	1.19	1.03	0.30	−1.12	3.57	−1.12	3.57
Gender	−0.07	0.17	−0.41	0.68	−0.41	0.27	−0.41	0.27
Age at primary intubation (months)	0.01	0.00	2.44	0.02	0.00	0.01	0.00	0.01
Reintubation	0.32	0.18	1.76	0.08	−0.04	0.68	−0.04	0.68
Emergency, planned intubation	0.47	0.23	2.10	0.04	0.03	0.92	0.03	0.92
History of tracheostomy	0.18	0.19	0.99	0.33	−0.18	0.55	−0.18	0.55
Mother’s pregnancy no.	0.00	0.05	−0.01	1.00	−0.09	0.09	−0.09	0.09
Birth weight, gram	0.00	0.00	−0.73	0.47	0.00	0.00	0.00	0.00
Height at birth, cm	0.02	0.03	0.62	0.54	−0.04	0.08	−0.04	0.08
Delivery type	−0.10	0.24	−0.44	0.66	−0.58	0.37	−0.58	0.37
Delivery category	−0.17	0.36	−0.47	0.64	−0.88	0.54	−0.88	0.54
Patient’s residence	−0.22	0.18	−1.22	0.22	−0.59	0.14	−0.59	0.14

**Table 5:** Spearman correlation analysis.

	Grade of Stenosis	Gender	Age at Primary Intubation (Months)	Reintubation	Emergency, Planned Intubation	History of Tracheostomy	Mother’s Pregnancy No.	Birth Weight, gram	Height at Birth, cm	Delivery Type	Delivery Category	Patient’s Residence
Grade of stenosis	1											
Gender	−0.032	1										
Age at primary intubation (months)	0.199	0.018	1									
Reintubation	0.106	−0.017	−0.127	1.000								
Emergency, planned intubation	0.206	0.020	0.102	−0.003	1.000							
History of tracheostomy	0.128	0.000	0.156	−0.023	0.118	1.000						
Mother’s pregnancy no.	0.036	−0.082	−0.164	0.043	0.102	0.156	1.000					
Birth weight, gram	−0.053	−0.013	0.061	−0.037	−0.055	0.088	−0.130	1.000				
Height at birth, cm	−0.031	−0.115	0.046	−0.046	−0.110	0.017	−0.035	0.799	1.000			
Delivery type	0.005	−0.031	−0.129	0.027	0.153	0.100	0.162	−0.354	−0.359	1.000		
Delivery category	−0.062	−0.069	0.064	−0.051	−0.080	0.025	−0.101	0.719	0.736	−0.440	1.000	
Patient’s residence	−0.091	0.010	0.158	0.070	−0.115	−0.110	−0.208	−0.032	−0.025	−0.006	0.071	1

## Data Availability

The raw data supporting the conclusions of this article will be made available by the authors upon request.
